# Supplementary material for: Predation and fragmentation portrayed in the statistical structure of prey time series
Source: BMC Ecol. 2009 May 6;9:10. doi: 10.1186/1472-6785-9-10 (PMC2689204; doi:10.1186/1472-6785-9-10)
Supplement: Additional file 2 — Voles and related classes ODDox Documentation. ODDox documentation of the agent-based model (ALMaSS) applied by Hendrichsen et al. The documentation is started by activating main.html. [file 1472-6785-9-10-S2.zip › Vole_ODDox/functions_func_0x75.html]

ALMaSS ODDox: Class Members - Functions

- Main Page
- Related Pages
- Classes
- Files

- Alphabetical List
- Class List
- Class Hierarchy
- Class Members

- All
- Functions
- Variables

- a
- b
- c
- d
- e
- f
- g
- h
- i
- k
- l
- m
- n
- o
- p
- r
- s
- t
- u
- v
- w
- ~

### - u -

- UnsetDirectFlag()
  : Vole\_Base
  , GeneticMaterial
  , GeneticMaterial1616- UnsetGeneticFlag()
    : Vole\_Base
    , GeneticMaterial
    , GeneticMaterial1616- UserDefinedFarm1()
      : UserDefinedFarm1- UserDefinedFarm10()
        : UserDefinedFarm10- UserDefinedFarm11()
          : UserDefinedFarm11- UserDefinedFarm12()
            : UserDefinedFarm12- UserDefinedFarm13()
              : UserDefinedFarm13- UserDefinedFarm14()
                : UserDefinedFarm14- UserDefinedFarm15()
                  : UserDefinedFarm15- UserDefinedFarm16()
                    : UserDefinedFarm16- UserDefinedFarm2()
                      : UserDefinedFarm2- UserDefinedFarm3()
                        : UserDefinedFarm3- UserDefinedFarm4()
                          : UserDefinedFarm4- UserDefinedFarm5()
                            : UserDefinedFarm5- UserDefinedFarm6()
                              : UserDefinedFarm6- UserDefinedFarm7()
                                : UserDefinedFarm7- UserDefinedFarm8()
                                  : UserDefinedFarm8- UserDefinedFarm9()
                                    : UserDefinedFarm9

---

Generated on Thu Jan 22 14:13:45 2009 for ALMaSS ODDox by 
 1.5.6
